# Effects of Traditional Chinese Acupuncture Compared with Sham Acupuncture on the Explosive Force Production by the Forearm Muscles in Female: A Randomized Controlled Trial

**DOI:** 10.1155/2021/1992753

**Published:** 2021-08-24

**Authors:** Shu Zhou, I-Lin Wang, Yi-Ming Chen, Rui Hu, Yu Su, Jia-Yu Shen, Jun Wang

**Affiliations:** ^1^College of Physical Education, Hubei Normal University, Huangshi 435002, Hubei, China; ^2^Jilin Sports University, No. 2476, Freedom Road, Nanguan District, Changchun City 130022, Jilin Province, China

## Abstract

**Background:**

Acupuncture can effectively enhance musculoskeletal rehabilitation, with effects such as reduced pain intensity and muscle tension and decreased disability.

**Objective:**

The purpose of this study was to determine the efficacy of traditional Chinese acupuncture (TCA) compared with sham acupuncture (SA) in explosive force production by the forearm muscles in females.

**Methods:**

A total of 32 subjects were included and randomly assigned to two groups: TCA group (*n* = 16): stimulated specific acupoints including Quchi (LI11), Shousanli (LI10), Hegu (LI4), Xiaohai (SI8), Tianjing (SJ10), and Waiguan (SJ5) for 15 minutes; SA group (*n* = 16): using superficial needle insertion at nonacupoints without stimulation. The subjects warmed up for the 3-time isokinetic test with an angular velocity of 30°/s and then performed a set of 15 full flexion (Flex) and extension (Ext) with an angular velocity of 180°/s using the CON-TREX isokinetic test training system recorded as the pretest. After acupuncture for 15 min, perform a set of the same isokinetic movement isokinetic records as the posttest. The average max torque, average work, average power, average peak power, average max speed, and total work were collected to evaluate the forearm explosive force changes. Use two-way repeated measures ANOVA to compare the difference before and after acupuncture between two groups.

**Results:**

The results showed that acupuncture conditions (sham acupuncture as well as true acupuncture) and the intervention times (not acupuncture or acupuncture for 15 min) have a significant interaction effect on forearm explosive force and joint stiffness (*P* < 0.05). The simple main effect showed that the selected parameters of the TCA group increased significantly after acupuncture (*P* < 0.05), while the SA group did not (*P* > 0.05). We speculate that the activation of muscle may be related to the selected acupuncture points.

**Conclusion:**

Acupuncture can produce excitation in motor nerves and muscles, and nerve stimulation increases the recruitment of motor units, thus improving the muscle explosive force.

## 1. Introduction

Acupuncture originated approximately 2,500 years ago in China and is most familiar to Western medicine as a complementary or alternative therapy. This treatment modality is still applied in its original form [1] and can be used in the clinical treatment of diseases. In addition to having a therapeutic effect, acupuncture can effectively enhance musculoskeletal rehabilitation, with effects such as reduced pain intensity and muscle tension and decreased disability. The addition of acupuncture to a treatment regimen may facilitate and enhance physiotherapy performance in musculoskeletal rehabilitation for tension neck syndrome. Furthermore, acupuncture increases alertness and promotes energetic feelings in patients with multiple sclerosis [2]. Thus, acupuncture is effective in reducing pain and rehabilitating various musculoskeletal conditions. Acupuncture has been applied as an enhancer of sports performance. The use of acupuncture in resistance and endurance sports activities has demonstrated the association of traditional acupuncture protocols with increased muscular strength and power [3]. Needling of specific acupuncture points produced an increase in physical performance capacity and improved regulation of heart rate and blood pressure [4]. Thus, acupuncture treatment not only improves athletic performance but also improves hemodynamics during endurance activities.

Acupuncture is able to produce the same excitatory characteristics within the motor nerves and muscles as exercise does [5]. Needles are inserted into specific points with nervous tissue. By stimulating these points with needles, the acupuncturist causes B-endorphins to be released for pain control [6]. Accordingly, acupuncture has been shown to reduce perceived pain arising from exercise-induced delayed-onset muscle soreness [7]. Acupuncture may enhance exercise performance and postexercise recovery. Unilateral electroacupuncture at selected acupoints even improved the muscle strength of both limbs [8]. Past research has found that overtraining and heavy sporting activity stress the immune status and neuroendocrine response of endurance athletes and lead to a decrease in overall sports performance [9]. However, acupuncture improved the subjective rating of muscle tension and fatigue and inhibited both the decrease in salivary secretory immunoglobulin and the increase in salivary cortisol after exercise stress [10]. Acupuncture has emerged as an alternative medical enhancer of human physical performance [3]. In a rat model, acupuncture can protect cells from acute sports injury, maintain the function of mitochondria to delay fatigue, prolong the working time of muscles, and delay muscle damage [11]. Therefore, this modality of treatment can increase physical function in both animals and humans. Electrical acupoint stimulation can enhance athletes' explosive strength. Specifically, the maximal peak moment of force, force, moment accelerating energy, and average power were increased [12]. In another study, transcutaneous electrical acupoint stimulation at selected acupoints enhanced the rate of muscle force recovery [13]. The treatment might also elevate the muscular pain threshold and change the perception of local muscle fatigue. The mechanism by which transcutaneous electrical acupoint stimulation influences recovery after exercise might be related to pain control [13]. Therefore, stimulation of the correct acupoints helps to delay exercise fatigue and improve muscle strength.

Isokinetic testing is considered the criterion standard for strength assessment. This method can be applied to the rehabilitation and prevention of throwing-related injuries and is important in determining return-to-play criteria [14]. Rehabilitation in medical clinics is also widely used. The energy efficiency of patients with chronic stroke is tested by measuring the isokinetic and isometric muscle strength of the upper limbs through the isokinetic test to achieve the effect of rehabilitation [15]. Acupuncture can achieve effective pain relief and improve the range of motion of joints [16]. Stimulating acupuncture points can improve athletic performance and improve biomechanical indexes, including maximum peak moment of force, force moment accelerating energy, and average power [12]. Combining acupuncture and isokinetic exercise training can be an effective rehabilitation therapy and may improve athletes' physical performance. At present, research on acupuncture extends beyond its use for rehabilitation therapy, but there is a lack of research regarding the effect of acupuncture on explosive force production by forearm muscles. Therefore, this study aims to explore the effect of acupuncture in explosive force production by the forearm muscles in females. Previous studies have found that acupuncture therapy has a direct effect on effectively improving the quadriceps muscle strength scale of recreational athletes [17]. The purpose of this study was to investigate the immediate effect of a single acupuncture session on strength improvement. None of the subjects in this study had previously received acupuncture with cumulative benefits.

## 2. Materials and Methods

### 2.1. Study Design

A randomised control trial (registration number: ChiCTR1900025407) was carried out to assess whether acupuncture has effects on explosive force production by the forearm muscles in females. The experiments were conducted at the Jilin Sport University Biomechanics Laboratory of the Health Technology College, Changchun, China, and was approved by the Joint Institutional Review Board of Jilin Sport University (JLSU; Changchun, China; JLSU-IRB no. 2018004). Volunteers were informed of the potential risks of acupuncture and were then asked to give written consent.

### 2.2. Subjects

In this study, the participants included 32 healthy female students (age = 25.4 ± 1.2 years, height = 161.6 ± 3.6 cm, body mass = 51.4 ± 5.4 kg, body mass index (BMI) = 19.6 ± 2.2 kg/m^2^) from Jilin Sport University volunteered to participate in this study (September 1, 2019, to September 30, 2019), and all subjects were randomly divided into the TCA group and SA group. There was no significant difference between subjects in the TCA group and the SA group in terms of height, weight, and age. Participants were recruited through a recruitment announcement. The exclusion criteria were as follows: stroke, severe heart disease, diabetes, neuromuscular disorders, inability to participate in physical activity, arrhythmia, use of antiarrhythmic drugs or a pacemaker, severe cardiovascular disease, current use of drugs that affect muscle mass or muscle performance, strenuous exercise or muscle soreness during the first 24 hours, obesity (BMI >22), and body weight of more than 90 kg.

### 2.3. Protocol

**Figure 1:**
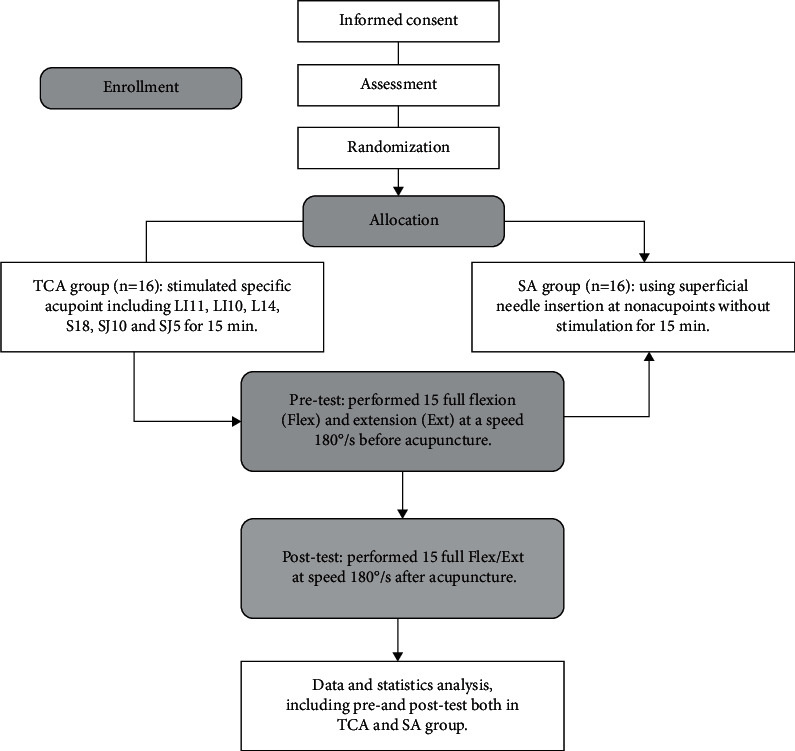
Flow diagram of the study.

The elbow Flex/Ext isokinetic measurements were performed using the CON-TREX isokinetic test training system (ConTrex MJ; CMV AG, Dübendorf, Switzerland). Subjects were tested separately. During the test, the angle between the seat back and the seat was adjusted to 85°; the seat was rotated 15° to the right, and the force measuring shaft was rotated to 15° to be parallel to the chair orientation. The subject sat in a comfortable position and was firmly fixed to the seat with the chest and waist straps and with the right elbow aligned with the axis of the dynamometer. The constant-velocity adapter was installed. The center of rotation of the elbow was carefully aligned with the center of rotation of the dynamometer's lever arm. Prior to isokinetic assessment, the subjects were performed a 3-time isokinetic test with an angular velocity of 30°/s for warm-up and to get familiar with the device. Then, the subject performed elbow joint Flex/Ext 15 times at 180°/s recorded as the pretest. After that, all subjects received 15-minute TCA or SA. After acupuncture, the subjects performed 15 times of full Flex/Ext with an angular velocity of 180°/s recorded as the posttest. A flow diagram of the protocol is shown in Figure 1.

### 2.4. Traditional Chinese Acupuncture and Sham Acupuncture

**Figure 2:**
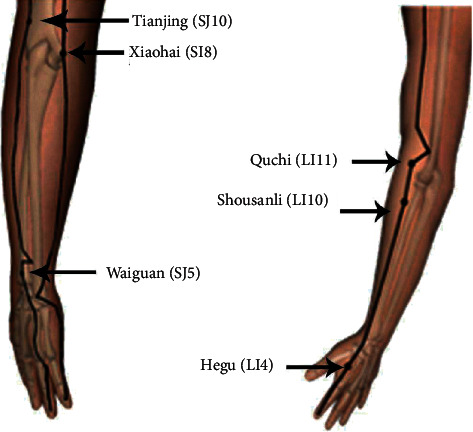
Acupuncture points.

Huatuo brand disposable sterile steel needles (size: 0.25 mm × 40 mm; manufactured by Suzhou Medical Appliance, Jiangsu, China) were used to stimulate the following acupuncture points: LI11, LI10, LI4, SI8, SJ10, and SJ5 (Figure 2). The needles were left in place for 15 min [18]. The acupuncturist placed his/her left index finger immediately above the acupoint, held the needle between his/her right thumb and index finger, and quickly pierced the skin of the patient. The slow inward pressure and twisting of the needle introduced a sensation of De Qi, radiating numb sensation, and acid bilge feeling. The acupuncturist then twisted the needle repeatedly at a speed of 3–5 r/s without the use of an electric or laser instrument. Each needle was rotated at 2 minutes, 5 minutes, and 10 minutes after insertion. While the needle was pulled out, sterilized cotton was pressed to the cheek for 5 s. The needle depths were approximately 50 mm [19].

A new sham acupuncture needle has been developed. Sham acupuncture points were used in a protocol similar to the traditional Chinese acupuncture points. For the sham condition, the same certified dental acupuncturist inserted the needles, which were left in place for 15 minutes, and during this period, use lifted, inserted, and twirled to stimulate the acupoints to generate the sense of “De Qi.” The needling experience for the SA group was the same as the TCA group, except the needles were in a nonacupuncture point and penetrated the skin only 2–4 mm.

### 2.5. Reasons for Acupoints Selection

The triceps are divided into the long head of the triceps, the lateral head, and the medial head, which are the main muscles for extension the elbow joint [20]. Brachioradial muscle can make forearm supination and flexion [21]. Studies have showed that acupuncture may increase the explosive forces generated by acupoint-related muscles by stimulating nerves [22]. In our study, SI8 and SJ10 are located in the triceps muscle and LI11 is located in the brachioradial muscle. Acupuncture points are selected to stimulate and may have similar effects.

### 2.6. Data Collection

The stiffness of the elbow joint was calculated using the following formula:

(1) $$\begin{array}{c} E_{\text{joint}}=\frac{\Delta M_{\text{joint}}}{\Delta \theta_{\text{joint}}}, \end{array}$$

where Δ*M*_joint_ is the change in the joint moment between the maximum elbow Ext and maximum elbow Flex, and Δ*θ*_joint_ is the angular displacement of the joint between maximum elbow Ext and maximum elbow Flex. The joint moment was normalized to the participant's body weight.

### 2.7. Data Analysis

The max torque values were identified in association with the max voluntary isometric contraction for the elbow Ext and at 180°/s isokinetic contraction for the elbow Flex. These values were used for the normalization of the other data. The max torque values were computed as the mean torque obtained in a 1 s window centered at the peak value. For each isokinetic set, the repetition showing the greatest torque was chosen for the analysis.

The data are reported as mean ± standard deviation (SD) and were analyzed with a mixed design two-way analysis of variance (ANOVA) with repeated measures. There were the between-subject factor group (sham acupuncture vs. true acupuncture) and within-subject factor (pretest and posttest). This design allowed for testing the main effect of groups, the main effect of time, and the interaction of groups by time. In case of significant interaction, simple effects were examined, i.e., the effects of one factor holding the other factor fixed. MATLAB software (version R2019a; MathWorks, Inc., Natick, MA) was used for the statistical analysis. *P* < 0.05 was defined as statistically significant. Calculate effect estimates (effect size: ES) to summarize the effects of acupuncture on each outcome by recalculation with a change score (e.g., posttest minus pretest) as the numerator and sample variability (e.g., standard deviation) as the denominator:

(2) $$\begin{array}{c} \text{ES}=M\left(\text{post}\right)-\frac{M\left(\text{pre}\right)}{\text{SD}}. \end{array}$$

Cohen categorized ES values as small (ES: 0.2 – 0.5), moderate (ES: 0.5 – 0.8), and large (ES: >0.8) [23].

## 3. Results

To detect differences between the intervention times (not acupuncture or acupuncture for 15 minutes) and both acupuncture conditions (sham acupuncture as well as true acupuncture), we performed a two-way ANOVA with the between-subject factor group (sham acupuncture vs. true acupuncture) and within-subject factor (pretest and posttest). Tables 1 and 2 summarize the results before and after acupuncture. For the intervention time and acupuncture conditions, a statistically significant interaction effect was shown in terms of all parameters (*P* < 0.05). Therefore, the results suggest that intervention time and acupuncture conditions did significantly affect the elbow joint explosive force and joint stiffness.

**Figure 3:**
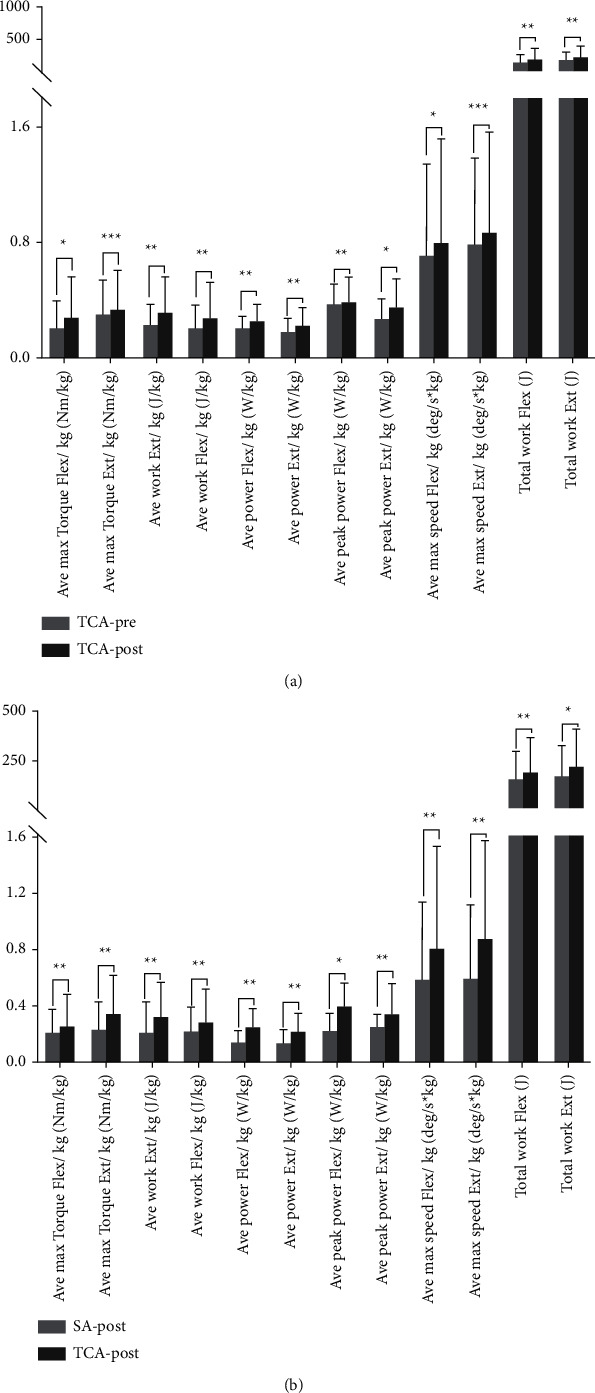
The differences of the elbow joint Flex/Ext for each isokinetic parameter within-subject factor (pretest and posttest) at the TCA group (a) and between-subject factor (TCA vs. the SA group) at the posttest (b). Note: values are mean ± SD. TCA, traditional Chinese acupuncture; SA, sham acupuncture. ^*∗*^*P* < 0.05. ^*∗∗*^*P* < 0.01. ^*∗∗∗*^*P* < 0.001.

At the end of the 15 min acupuncture, further analysis of the simple main effect revealed a significant difference between the SA and TCA groups across time. There was significant difference in the TCA groups before and after intervention, respectively, that significantly increased after acupuncture, including the average max torque Flex/Ext (+Δ = 0.41, ES = 0.58, *P*=0.035 and +Δ = 0.13, ES = 1.21, *P* < 0.001), average work Flex/Ext (+Δ = 0.88, ES = 0.50, *P*=0.003 and +Δ = 0.41, ES = 0.88, *P*=0.003), average power Flex/Ext (+Δ = 0.31, ES = 0.87, *P*=0.003 and +Δ = 0.24, ES = 0.77, *P*=0.007), average peak power Flex/Ext (+Δ = 0.09, ES = 0.86, *P*=0.004 and +Δ = 0.32, ES = 0.78, *P*=0.007), average max speed Flex/Ext (+Δ = 0.13, ES = 0.62, *P*=0.026 and +Δ = 0.34, ES = 1.48, *P* < 0.001), and total work Flex/Ext (+Δ = 0.35, ES = 1.04, *P*=0.001 and +Δ = 0.34, ES = 0.99, *P*=0.002). However, in the SA group, there was no significant difference in those same parameters before and after acupuncture or after acupuncture compared with the TA group (all *P* > 0.05) (Figure 3(a)).

Additionally, there were significant differences between the TA group and the SA group after acupuncture. Specifically, significant differences were found between the TCA group and the SA group after acupuncture, including the average max torque Flex/Ext (+Δ = 0.28, ES = 1.07, *P*=0.005 and +Δ = 0.34, ES = 01.34, *P*=0.001), average work Flex/Ext (+Δ= 0.36, ES = 1.11, *P*=0.004 and +Δ= 0.32, ES = 0.99, *P*=0.009), average power Flex/Ext (+Δ= 0.70, ES = 1.08, *P*=0.005 and +Δ = 0.55, ES = 1.03, *P*=0.007), average peak power Flex/Ext (+Δ= 0.65, ES = 0.89, *P*=0.017 and +Δ= 0.58, ES = 1.10, *P*=0.004), average max speed Flex/Ext (+Δ= 0.35, ES = 1.34, *P*=0.001 and +Δ= 0.42, ES = 1.29, *P*=0.001), and total work Flex/Ext (+Δ= 0.24, ES = 1.34, *P*=0.006 and +Δ= 0.27, ES = 0.93, *P*=0.014) (Figure 3(b)).

**Figure 4:**
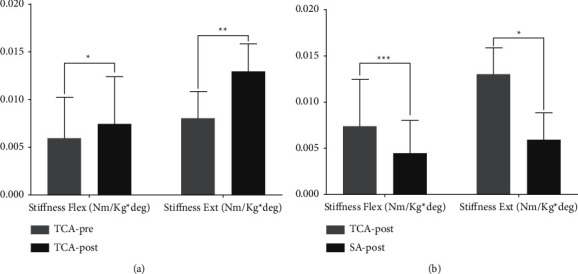
The differences of elbow joint stiffness Flex/Ext within-subject factor (pretest and posttest) at the TCA group (a) and between-subject factor (TCA vs. the SA group) at the posttest (b). Note: values are mean ± SD. TCA, traditional Chinese acupuncture; SA, sham acupuncture. ^*∗*^*P* < 0.05. ^*∗∗*^*P* < 0.01. ^*∗∗∗*^*P* < 0.001.

Figure 4 shows the joint stiffness of elbow joint Flex/Ext for the TCA group and SA group as assessed before and after intervention. There were significant differences before and after acupuncture at the TCA group, that is, stiffness Flex (+Δ= 0.11, ES = 0.70, *P*=0.013) and stiffness Ext (+Δ= 0.50, ES = 0.75, *P*=0.009) (Figure 4(a)). After acupuncture, the stiffness Flex/Ext also significantly and respectively increased compared with the SA group (+Δ= 0.43, ES = 1.38, *P* < 0.001 and+Δ= 0.80, ES = 0.53, *P*=0.031) (Figure 4(b)).

Accordingly, after 15-min acupuncture at LI11, LI10, LI4, SI8, SJ10, and SJ5 can immediately improve the explosive force and joint stiffness of the elbow joint Flex/Ext compared to sham acupuncture.

## 4. Discussion

After acupuncture, the average max torque Flex/Ext value was greater than the pretest values, which may be due to increased muscle strength and increased explosive power. Neural factors are an important determinant of torque gain in this training protocol [24]. The neural effects of acupuncture may stimulate muscle contraction, increasing the muscles explosive force during training or competition. The mild tonic somatosensory stimulation produced by acupuncture produces long-term plastic changes in the excitability of very distant nervous structures that exert motor control over remote muscles [25]. Enhanced muscle fiber conduction velocity increases torque [26, 27]. Therefore, it is possible to increase the speed of muscle fiber conduction velocity through acupuncture, causing the average max torque Flex/Ext to improve. The phenomenon of related physiological reactions after acupuncture is called “De Qi” and is widely considered necessary for the therapeutic effect of acupuncture. “De Qi” can increase the torque and muscle mass of the upper and lower extremities [28]. Transcutaneous electrical acupoint stimulation of muscles that have developed fatigue has been found to increase the rate of muscle force recovery and peak torque. Past research has shown that acupoint stimulation (true ST 36) in young football players leads to increased knee extension and flexion strength and increased peak torque [29]. After stimulation of the LI11, LI10, LI4, SI8, SJ10, and SJ5 acupoints, the average max torque Flex/Ext was improved compared with the pretest, suggesting that acupuncture can stimulate the muscles and increase the torque.

In our study, we found that the average max speed Ext and the max speed Ext were significantly increased after acupuncture. Additionally, the total work Ext increased compared with the pretest value. Past research has found that electrical acupoint stimulation increases the maximal peak moment, force, moment accelerating energy, and average power [12]. In a study of astronauts, stimulating the antagonist muscle to resist the volitional contraction of the agonist increased the extension torque, average power, and total work output of the elbow joint [30]. Therefore, acupuncture may increase the speed of muscle contraction and increase work output. Acupuncture in rats can increase Ca^2+^-ATPase (adenosine triphosphate, ATP) activity and Ca^2+^ content in the sarcoplasmic reticulum of skeletal muscle cells in a state of motor fatigue, which may protect cells from acute sport injury and maintain the function of mitochondria to delay fatigue, prolong the working time of muscles, and protect muscles from damage; this effect on Ca^2+^ transport may contribute to the beneficial effect of acupuncture on motor ability [11,31]. Therefore, the increase in total work Ext in this study may occur because acupuncture stimulates muscles to increase their sarcoplasmic reticulum Ca^2+^ content, stimulates Ca^2+^-actin interactions, and increases the work output of the muscles during contraction.

In this study, we found that elbow joint stiffness was greater after acupuncture. Acupuncture is able to induce long-term plastic changes in the central nervous system [25]. Increasing the conduction velocity of muscle fibers increases the torque at the corresponding joint [26,27]. Past research has shown that acupuncture stimulation enhances stiffness and improves the viscoelasticity of tendon structures [32]. Muscle contractions preceding an activity can result in increased force generation through PAP. Isometric muscular contractions may affect subsequent strength and power performance. Therefore, acupuncture may improve neuromuscular control and generate nervous system excitation, thus increasing joint stiffness.

## 5. Conclusion

This study focused on the changes in explosive force production by the forearm muscles in females before and after acupuncture both in TCA and SA groups. Our data show that 15-minute acupuncture is beneficial to the production of forearm muscles by inducing the PAP effect. The choice of acupoints also affects the difference in elbow joint stiffness upon extension and flexion. Acupuncture can produce excitation in motor nerves and muscles, and nerve stimulation increases the recruitment of motor units, thus improving the muscle explosive force (such as torque, power, and work). This experiment is a clinically valuable contribution to motor neuromuscular therapy, providing a reference to a method for coaches to improve athletic performance.

This study has several limitations to consider. First, only articles written in English were included, which may limit the scope of some of the acupuncture literature. In addition, the present study included only young healthy females; therefore, compared with athletes using the same research methods, the results may not be the same. Furthermore, future research will explore the timeliness and gender differences of acupuncture that improve the explosive force of the forearm muscles of the candidates.

## Figures and Tables

**Table 1:** Mean ± SD of the elbow joint flexion (Flex) muscles before and after acupuncture for each variable.

Characteristic	Treatment	Pre	Post	Delta	*P* values		
					Main effects (time)	Main effects (group)	Interaction (time ^*∗*^ group)
Average max torque Flex/kg (nm/kg)	SA	0.35 ± 0.10	0.32 ± 0.08	−0.09	0.469	0.058	0.033^*∗*^
	TCA	0.34 ± 0.07	0.41 ± 0.09	0.41			
Average work Flex/kg (J/kg)	SA	0.34 ± 0.11	0.36 ± 0.08	0.06	0.004	0.058	0.021^*∗*^
	TCA	0.33 ± 0.13	0.49 ± 0.15	0.48			
Average power Flex/kg (J/kg)	SA	0.19 ± 0.09	0.20 ± 0.08	0.05	0.012	0.018	0.040^*∗*^
	TCA	0.26 ± 0.15	0.34 ± 0.16	0.31			
Average peak power Flex/kg (W/kg)	SA	0.31 ± 0.16	0.31 ± 0.15	0.00	0.007	0.031	0.001^*∗*^
	TCA	0.47 ± 0.27	0.51 ± 0.28	0.09			
Average max speed Flex/kg (deg/s^*∗*^kg)	SA	1.02 ± 0.29	0.97 ± 0.21	−0.05	0.238	0.009	0.023^*∗*^
	TCA	1.16 ± 0.27	1.13 ± 0.29	0.13			
Total work Flex (J)	SA	236.94 ± 61.23	253.96 ± 56.19	0.07	0.001	0.087	0.019^*∗*^
	TCA	234.32 ± 58.00	315.49 ± 62.46	0.35			
Stiffness Flex (Nm/kg^*∗*^deg)	SA	0.008 ± 0.003	0.007 ± 0.002	−0.13	0.763	0.005	0.027^*∗*^
	TCA	0.009 ± 0.003	0.011 ± 0.004	0.11			

Note: values are mean ± SD. TCA, traditional Chinese acupuncture; SA, sham acupuncture. ^*∗*^There are significant differences in the interaction between group and time (*P* < 0.05).

**Table 2:** Mean ± SD of the elbow joint extension (Ext) muscles before and after acupuncture for each variable.

Characteristic	Treatment	Pre	Post	Delta	*P* values		
					Main effects (time)	Main effects (group)	Interaction (time ^*∗*^ group)
Average max torque Ext/kg (nm/kg)	SA	0.37 ± 0.10	0.37 ± 0.09	0.00	0.034	0.004	0.005^*∗*^
	TCA	0.47 ± 0.14	0.53 ± 0.14	0.13			
Average work Ext/kg (J/kg)	SA	0.31 ± 0.08	0.34 ± 0.10	0.10	0.001	0.047	0.028^*∗*^
	TCA	0.32 ± 0.09	0.45 ± 0.11	0.41			
Average power Ext/kg (J/kg)	SA	0.22 ± 0.10	0.20 ± 0.07	−0.09	0.229	0.060	0.032^*∗*^
	TCA	0.25 ± 0.13	0.31 ± 0.13	0.24			
Average peak power Ext/kg (W/kg)	SA	0.34 ± 0.22	0.31 ± 0.21	−0.09	0.071	0.084	0.002^*∗*^
	TCA	0.37 ± 0.43	0.49 ± 0.49	0.32			
Average max speed Ext/kg (deg/s^*∗*^kg)	SA	1.00 ± 0.25	0.96 ± 0.22	−0.12	0.178	0.005	0.030^*∗*^
	TCA	1.21 ± 0.36	1.36 ± 0.38	0.34			
Total work Ext (J)	SA	250.18 ± 67.47	280.10 ± 70.81	0.12	<0.001	0.073	0.037^*∗*^
	TCA	264.75 ± 84.25	355.35 ± 90.63	0.34			
Stiffness Ext (Nm/kg^*∗*^deg)	SA	0.011 ± 0.009	0.008 ± 0.004	−0.27	0.334	0.261	0.009
	TCA	0.010 ± 0.006	0.015 ± 0.011	0.50			

Note: values are mean ± SD. TCA, traditional Chinese acupuncture; SA, sham acupuncture. ^*∗*^There are significant differences in the interaction between group and time (*P* < 0.05).

## Data Availability

The datasets used and analyzed to support the findings of this study are included within the article.
